# Deazaneplanocin A Is a Promising Drug to Kill Multiple Myeloma Cells in Their Niche

**DOI:** 10.1371/journal.pone.0107009

**Published:** 2014-09-25

**Authors:** Jérémie Gaudichon, Francesco Milano, Julie Cahu, Lætitia DaCosta, Anton C. Martens, Jack-Michel Renoir, Brigitte Sola

**Affiliations:** 1 Equipe Associée 4652, Université de Caen, Normandie Univ, Caen, France; 2 Institut National de la Santé et de la Recherche Médicale U749, Institut Gustave Roussy, Villejuif, France; 3 Department of Immunology, University Medical Center Utrecht, Utrecht, The Netherlands; University of Maryland School of Medicine, United States of America

## Abstract

Tumoral plasma cells has retained stemness features and in particular, a polycomb-silenced gene expression signature. Therefore, epigenetic therapy could be a mean to fight for multiple myeloma (MM), still an incurable pathology. Deazaneplanocin A (DZNep), a S-adenosyl-L-homocysteine hydrolase inhibitor, targets enhancer of zest homolog 2 (EZH2), a component of polycomb repressive complex 2 (PRC2) and is capable to induce the death of cancer cells. We show here that, in some MM cell lines, DZNep induced both caspase-dependent and -independent apoptosis. However, the induction of cell death was not mediated through its effect on EZH2 and the trimethylation on lysine 27 of histone H3 (H3K27me3). DZNep likely acted through non-epigenetic mechanisms in myeloma cells. *In vivo*, in xenograft models, and *in vitro* DZNep showed potent antimyeloma activity alone or in combination with bortezomib. These preclinical data let us to envisage new therapeutic strategies for myeloma.

## Introduction

Multiple myeloma (MM) is a hematological malignancy characterized by the accumulation of abnormal plasma cells in the bone marrow. MM is the second most-common hemopathy and represents 1% of all cancers. Despite the emergence of new drugs including immunomodulators (lenalinomide) and proteasome inhibitors (bortezomib) that have significantly extended patients’ survival, this disease remains incurable, with severe complications, and always leads to death [1]. This explains the need of new drugs and/or therapeutic strategies. The involvement of epigenetic alterations in oncogenesis starts to be well understood. In turn, epigenetic therapies have emerged and seemed efficient in the treatment of some hemopathies including MM [2].

The polycomb repressive complexes (PRC) are key mediators of transcriptional repression. PRC2 controls the pivotal methylation of lysine 27 of histone H3 (H3K27) catalyzed by the SET-domain containing enhancer of zest homolog 2 (EZH2) protein and its cofactors. Components of PRC2 are required for embryonic development and notably loss of *EZH2* gene is associated with a block in B- and T-cell differentiation [3]. Moreover, *EZH2* acts as an oncogene, is overexpressed in many solid cancers and lymphomas, in both advanced and metastatic diseases [4].

In a subtype of diffuse large B-cell lymphomas and follicular lymphomas, heterozygous missense mutations at Y641, within the SET domain of *EZH2* have been described [5], [6]. This mutation results in gain-of-function as the expression of the mutated allele adds up to the wild type one and increases level of H3K27me3 [7], [8]. Although such mutations have not been reported so far in MM, *EZH2* is clearly overexpressed in MM cells and contributes to cell survival [9]. This is consistent with data reporting the enrichment for H3K27me3 marked genes [10] as well as the finding of prevalent mutations of the H3K27-demethylase UTX [11] in MM cells. Although the functional role of EZH2 in maintaining the survival of MM cells is unknown, it has been shown that depletion of EZH2 could trigger apoptosis. This was achieved using the 3-deazaneplanocin A (DZNep) *in vitro* on MM cell lines [10], [12]. DZNep is an inhibitor of S-adenosyl-L-homocysteine (AdoHcy) hydrolase, the enzyme responsible for the reversible hydrolysis of AdoHcy to adenosine and homocysteine within the methionine cycle. Its inhibition by DZNep leads to the accumulation of AdoHcy and, in turn, *EZH2* downregulation [4]. The depletion of EZH2 and H3K27me3 triggers the apoptosis of cancer cells [13], [14]. We analyzed here the effects of DZNep on MM cell lines and investigated its mode of action. We then determined the efficacy of DZNep *in vivo* by using xenograft models. Collectively, our data showed that DZNep could be effective to treat some severe forms of MM.

## Materials and Methods

### Chemicals, siRNAs and antibodies

Quinoyl-valyl-O-methylaspartyl-(2,6-difluoro-phenoxy)-methyl ketone or Q-VD-OPh, everolimus, propidium iodide (PI), cycloheximide (CHX) were purchased from Sigma-Aldrich (Saint-Quentin Fallavier, France), LY294002 from Biomol (Hamburg, Germany), bortezomib from Selleckchem (Houston, TX), MG-132 from Calbiochem (Gibbstown, NJ), DZNep from Cayman Chemical, (Ann Arbor, MI). Drugs were dissolved in ethanol (EtOH) or DMSO to obtain stock solutions (10–50 mM) and were diluted in serum-free culture medium before use. For control experiments using drugs, ethanol (EtOH) or dimethylsulfoxide (DMSO) were added as vehicles at the same concentration. For *in vivo* experiments, DZNep was dissolved in 10% D-mannitol (Sigma-Aldrich), then diluted at the appropriate concentration in PBS to reach 0.1% D-mannitol for i.p mice injections.

The following antibodies (Abs) were used in the study: anti-β-actin (sc-47778), anti-caspase 3 (sc-7148), and anti-caspase 8 (sc-7890) from Santa Cruz Biotechnologies (Santa Cruz, CA); anti-caspase 9 (#9508), anti-poly (ADP-ribose) polymerase or PARP (#9542) and anti-EZH2 (#3147) from Cell Signaling Technology (Danvers, MA); anti-B-cell lymphoma 2 or BCL2 (110887) from Dako (Courtaboeuf, France), anti-glyceraldehyde-3-phosphodeshydrogenase (GAPDH, #4300) from Applied Biosystems/Ambion (Austin, TX) and anti-H3K27me3 (mAb6002) from Abcam (Paris, France).

### Cell cultures and cell viability determination

EJM (ACC-560), JJN3 (ACC-541), L363 (ACC-49) and RPMI 8226 (here 8226, ACC-402) cells were purchased from DSMZ (Leibniz Institute, German Collection of Microorganisms and Cell cultures, Braunschweig, Germany); LP1 [15], NCI-H929 (here H-929) [16], U266 [17] cells were provided by R Bataille (CRCNA research facility, Nantes, France) and OPM2 [18] cells from D Bouscary (Institut Cochin, Paris, France). The identity of non-commercially obtained cells was checked as described before [19]. Multiple myeloma cell lines (MMCLs) were maintained in RPMI 1640 medium supplemented with 100 U/mL penicillin, 100 U/mL streptomycin, 2 mM L-glutamine (all from Lonza, Basel, Switzerland) and 10% fetal calf serum (FCS, PAA Lab., Villacoublay, France). For cell viability studies, exponentially growing cells were seeded at a density of 2×10^5^ cells/mL into six-well plates in 0.1% DMSO-containing medium or various concentrations of DZNep. The human stromal cell line HS-5 obtained from ATTCC (CRL-11882, Manassas, VA) was maintained in Dulbecco’s modified eagle’s medium containing antibiotics, L-glutamine and 10% FCS. Viable cells, excluding Trypan blue, were counted in a hemocytometer at various time intervals after various drug concentration treatments. The 8226 tumoral population contains both CD138^low^ and CD138^high^ cells; they were separated using anti-CD138 Ab coupled to magnetic microbeads (ref. 130-051-301, Miltenyi Biotec, Bergisch Gladbach, Germany) according to [20].

### Cell proliferation determination

Cell proliferation was assayed using a ((2,3-bis(2-methoxy-4-nitro-5-sulfophenyl)-2H-tetrazolium-5-carboxanilide) inner salt) MTS assay, here the CellTiter 96 AQueous One Solution Cell Proliferation assay (Promega, Charbonnières, France). According to the supplier, 10^4^ cells were seeded into 96-well plates, incubated with vehicle or various concentrations of drug for 24–144 h. Each culture condition was realized at least in triplicate. The absorbance values at 492 nm were corrected by subtracting the average absorbance from the control wells containing “no cells”.

### Cell cycle analysis, apoptosis determination and reactive oxygen species detection by flow cytometry sorting

For each culture condition, cultured cells were washed and suspended in ice-cold EtOH (80% in phosphate buffered saline, PBS). EtOH-fixed cells were then suspended in PBS containing 100 µg/mL RNase A (Roche Molecular Biochemicals, Meylan, France) and 20 µg/mL PI then incubated for 30 min before fluorescence-activated cell sorting (FACS) analysis. For apoptosis determination, cells were suspended in 100 µL of PBS containing 10 µL of anti-APO2.7 Ab (IOTest, Beckman Coulter) and incubated for 30 min at room temperature before FACS analysis. For the detection of reactive oxygen species (ROS), 5-(and-6)-chloromethyl-2′,7′-dichlorodihydrofluoresceindiacetate acetyl ester or CM-H_2_DCFDA (C6827, Invitrogen) staining was performed according to the manufacturer’s instructions. On average, 2×10^4^ cells were analyzed with a Gallios cytometer (Beckman Coulter) and data were analyzed with the Kaluza software (Beckman Coulter).

### Morphological analyses of MMCLs

Cells were treated with either DZNep or vehicle, cytospun and stained with May-Grünwald-Giemsa before observation and image acquisition. A systematic uniform sampling was used in order to cover the whole slide (space between two acquisitions: 1 mm). Each image was acquired thanks to a microscope (AX70, Olympus, objective 40x) coupled to a camera (SpOt RT/KE, Diagnostics Instruments). The true-color images (24 bits RGB color) were saved in uncompressed tagged image file format (.tif) with a resolution of 1600×1200 pixels. A script was written in Python language with the specific module Mahotas to segment each cell. This script was based on color deconvolution [21] and mathematical morphology [22]. Only integral cells were kept. An interactive step was included after image processing for eliminating potential errors (bad segmentation, artifacts elements, etc.). For each cell, the perimeter, area and circularity (defined as (perimeter)^2^/4×π×area) were computed. The circularity parameter allows objectivizing the shape of an object (C = 1 when the object is a circle, C = 0 when the object is a line). For transmission electronic microscopy, cells were prepared and examined with a JEOL 2011 as previously described [19].

### Western blots

Whole cell lysates were obtained by incubating cells for 30 min on ice in the following buffer: 1% NP-40, 100 mM Tris-HCl (pH 7.4), 5 mM EDTA, 150 mM NaCl and a cocktail of protease inhibitors (cOmplete EDTA-free, Roche Diagnostics, Meylan, France). Proteins (50 µg) were separated by SDS/PAGE and blotted onto nitrocellulose membranes (Bio-Rad). Membranes were incubated with various Abs then exposed to a chemiluminescence detection reagent (Western Lightning Plus, PerkinElmer, Waltham, MA).

### Real-time qRT-PCR

According to supplier’s instruction, RNA samples were extracted with TRIzol reagent (Ambion, Austin, TX) from cultured MM cells. Total RNA was reverse-transcribed by using M-MuLV reverse transcriptase with random primers (all from Invitrogen, Life Technologies, Saint-Aubin, France). Real-time qRT-PCR was performed with a thermocycler (StepOne Plus, Applied Biosystems) using the GoTaq Master Mix (Promega). The relative expression of each gene was quantified by the comparative threshold (C_t_) method (ΔΔC_t_) by using *RPLP0* gene as internal control with the StepOne v2.2.2 (Applied Biosystems) software. Specific primers produced by Eurogentec (Liege, Belgium) were the following: *EZH2*, F 5′-ATG ATG GAG ACG ATC CTG AA-3′, R 5′-TCT TCT GCT GTG CCC TTA-3′; *SUZ12*, F 5′-AAA CGA AAT CGT GAG GAT GG-3′, R 5′-CCA TTT CCT GCA TGG CTA CT-3′; *EED*, F 5′-ATC CGG TTG TTG CAA TCT TA-3′, R 5′-TTT GGA TCT CTT GGA TGG AA-3′; *BMI1*, F5'-CTG GTT GCC CAT TGA CAG C-3′, R 5′-CAG AAA ATG AAT GCG AGC CA-3′; *RPLP0*, F 5′-CCA GGC GTC CTC GTG GAA GTG-3′, R 5′-TTC CCG CGA AGG GAC ATG CG-3′.

### Xenograft models

Experiments were conducted in accordance with the recommendations of EEC (86/609/CEE). Experiments were approved by the Animal Experimental Ethics Committee of our Institution (Institut Gustave Roussy, France, permit n°26-2012-13). We used the RPMI 8226-Luc-GFP cell model described previously [23]. Six-week old non-obese diabetic/severe combined immunodeficiency/interleukin 2 receptor γ chain −/− or NSG mice were injected in the caudal vein with 5×10^6^ MM cells. Three days later, mice received either vehicle (n = 5) or were injected i.p. with 3 mg/kg DZNep twice a week (n = 5) until the end of the experiment. In a second series, using the same protocol of cells injection and engraftment, treatments started ten days later. NSG mice received vehicle (n = 5), bortezomib 0.4 mg/kg twice a week (n = 5), DZNep 1.5 mg/kg every two days (n = 5) or bortezomib in combination with DZNep 1.5 mg/kg every two days (n = 5). For bioluminescence imaging (BLI), mice were injected with 75 mg/kg of D-luciferine (Promega) and then analyzed with a Xenogen Optical In Vivo System (IVIS) coupled to Living Image Acquisition and Analysis software (Perkin Elmer, Waltham, MA). Ventral plus dorsal luminescence were determined by quantifying photon flux through the whole mouse and quantified. Mice were imaged at successive time points and were euthanized at the end of the treatment.

### Tumor phenotyping by immunohistochemistry

At the end of experiments, bones were excised and immediately fixed in 4% paraformaldehyde (Electron Microscopy Sciences) prior to dehydration and paraffin inclusion for hematoxylin/eosin staining (HES) and immunohistochemistry (IHC). Acquired digital images of whole histological tumor sections were recorded using a Nikon SuperCoolscan 8000 ED slide scanner equipped with a FH-8G1 medical slide holder (Nikon, Champigny-sur-Marne, France). IHC analyzes were realized using a rabbit polyclonal Ab against phospho-H3 (p-H3, 1/50, #9701, Cell Signaling Tech.), a marker of M phase or against cleaved caspase 3 (cl. caspase 3, 1/100, #9661S, Cell signaling Tech.), a marker of apoptosis according to [24]. N-Histofine Simple Stain anti-rat and N-Histofine anti-mouse stain kits (both from Nichirei, Japan) were used for CD34 and CD138 detection, respectively. For each antibody, isotype control was included in each experiment. Micrograph images were obtained either at x100 or x630 magnification with immersion objective using a Zeiss Axiophot microscope (Carl Zeiss, Oberkochen, Deutschland).

### Statistical analysis

Student’s *t*-test was used to determine the significance of differences between two experimental groups. Data were analyzed with a two-sided test and *p*<0.05 (*) was considered significant.

## Results

### DZNep induces myeloma cells death

**Figure 1:**
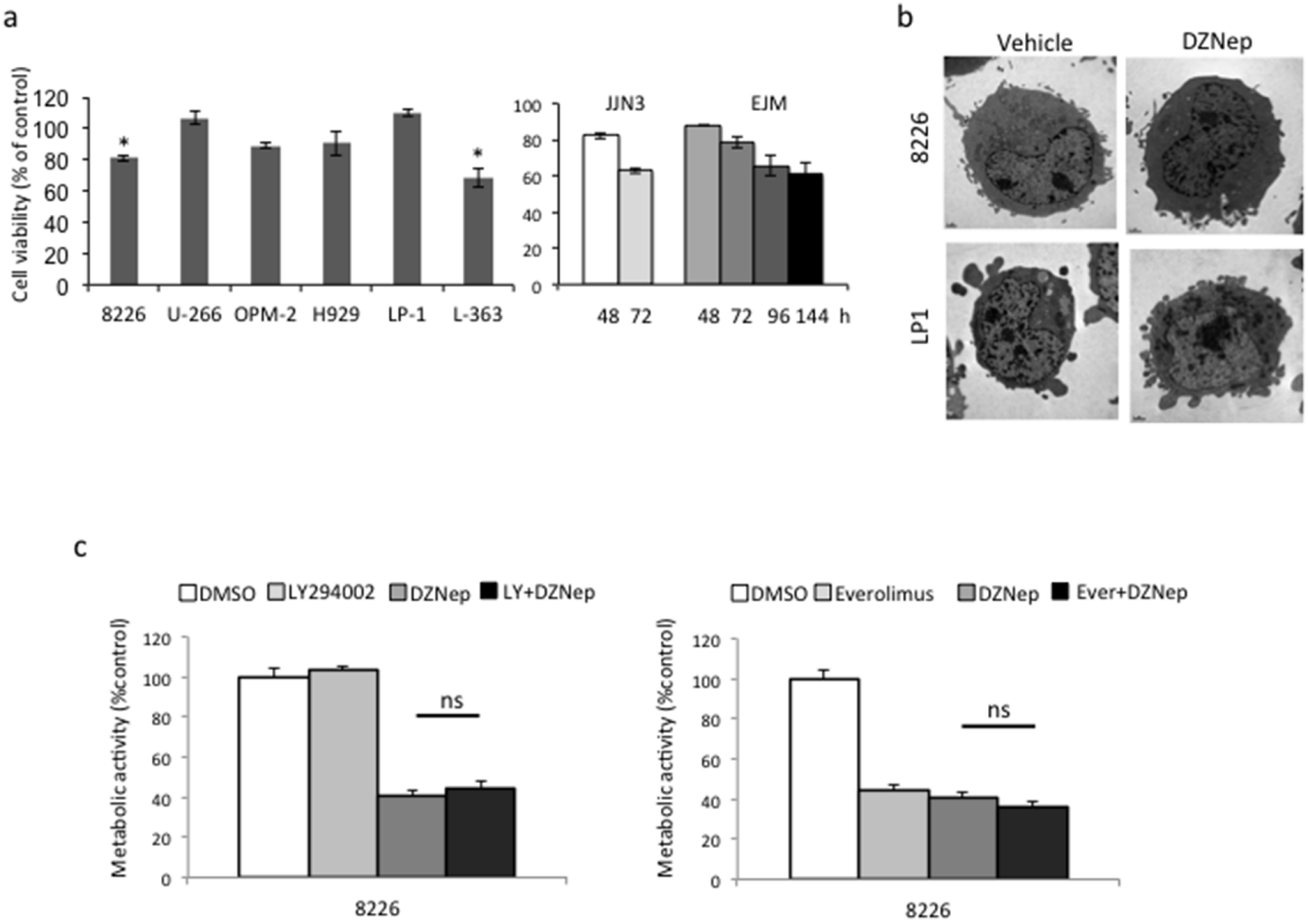
DZNep induces myeloma cells death. Exponentially growing MM cells were either treated with vehicle (DMSO 0.1%) or DZNep 1 µM for 72 h (**a left part**) or 48–144 h (**a right part**). (**a**) Cell viability was assessed by Trypan blue exclusion. The percentage of viable cells referred to control experiments assigned to 100%. The experiment has been repeated three times, histograms show means ± SD, *p<0.05. (**b**) Responsive 8226 and resistant LP1 cells were either treated with vehicle or DZNep (1 µM for 24 h) then examined by transmission electronic microscopy. (**c**) RPMI 8226 cells were treated with vehicle, the PI3K inhibitor LY94002 (1 µM for 24 h, **left part of the figure**), the mTOR inhibitor everolimus (10 nM for 24 h, **right part of the figure**), DZNep (1 µM for 24 h) or both and cell proliferation assayed by a MTS assay. For each culture condition, cells were seeded in three to five wells. The experiments have been repeated two or three times. A representative experiment is shown with the mean and SD values. ns, not significant.

To study the effects of DZNep on cell growth and survival, we tested various DNZep concentrations (0.1, 0.5 and 1 µM) on eight MMCLs: 8226, U266, OPM2, H929, LP1, JJN3, EJM and L363 for several time intervals (24 to 144 h). DZNep-treated cells were analyzed for Trypan blue exclusion and compared to vehicle-treated cells. Four cell lines (U266, OPM2, H929 and LP1) were resistant to the treatment whereas the other four (8226, L363, JJN3 and EJM) were sensitive. As exemplified Figure 1a, a 1 µM- and 72 h-treatment was cytotoxic on 8226 and L363 (loss of viability of 19% and 31%, respectively). JJN3 cells were also sensitive to DZNep (loss of viability of 37% at 72 h, Figure 1a). However, because of a slower proliferation rate compared to other MMCLs, a loss of viability of 31% was reached 144 h post-treatment for EJM cells (Figure 1a). We next analyzed the proliferation capacity of cells treated with the same concentrations of DZNep and the same time intervals using a MTS assay. Again, we observed a time- and dose-dependent decrease of cell proliferation for 8226, L363 and JJN3 cells (data not shown). This assay allowed us to calculate the half maximal inhibitory concentration (IC_50_) at 72 h: 1 µM for 8226, 0.7 µM for L363, and 1.1 µM for JJN3. Our results showed that, in good agreement with previous reports [10], [12], DZNep was efficient to induce the death of MM cells.

### DZNep-induced cell death is not autophagy or necroptosis

Since autophagy was recognized as a regulator of both cell viability and death in MM [25], we looked for markers of autophagy in DZNep-treated 8226 and LP1 (as control) cells. As shown Figure 1b, in treated cells, we detected neither autophagosomes nor multilamellar bodies. The absence of autophagic cell death was further confirmed by the inhibition of the phosphoinositide 3-kinase (PI3K)/AKT/mammalian target of rapamycine (mTOR) pathway that controls autophagy in MM cells [26]. The pretreatment of 8226 cells with LY294002 (an inhibitor of PI3K/AKT, 1 µM for 2 h) or everolimus (an inhibitor of mTOR, 10 nM for 2 h) did not modify the response towards DZNep (Figure 1c).

**Figure 2:**
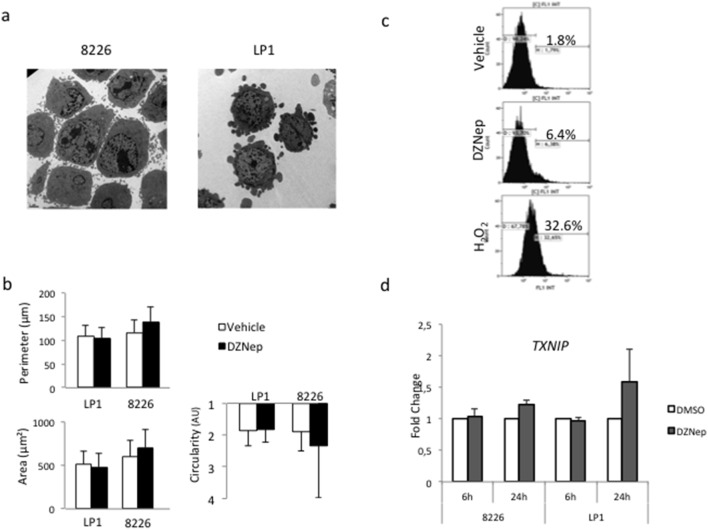
DZNep-induced cell death is not necroptosis. (**a**) Responsive 8226 and resistant LP1 cells were either treated with vehicle or DZNep (1 µM for 24 h) then examined by transmission electronic microscopy. (**b**) Responsive 8226 and resistant LP1 cells were either treated with vehicle or DZNep (1 µM for 24 h). The perimeter (in µm), the area (in µm^2^) and the circularity (in arbitrary unit) of vehicle-(in white) or DZNep-treated (in black) LP1 and 8226 cells were determined by image analysis as described in the method section. (**c**) L363 cells were treated with vehicle, DZNep 1 µM for 24 h, or hydrogen peroxide (H_2_O_2_), incubated with CM-H_2_DCFDA before FACS analysis. At least, 2×10^4^ events were gated. The percentage of stained cells (*i.e*. cells producing ROS) is indicated on the graph. (**d**) Responsive 8226 and resistant LP1 cell lines were treated for 6 or 24 h with DZNep 1 µM. The transcriptional expression of *TXNIP* was determined by qRT-PCR using the ΔΔC_t_ method with *RPLP0* as internal standard. The fold change was calculated as 2^−ΔΔ*Ct*^. Indicated values corresponded to the mean ± SD from at least three independent experiments done with two distinct RNA preparations.

**Figure 3:**
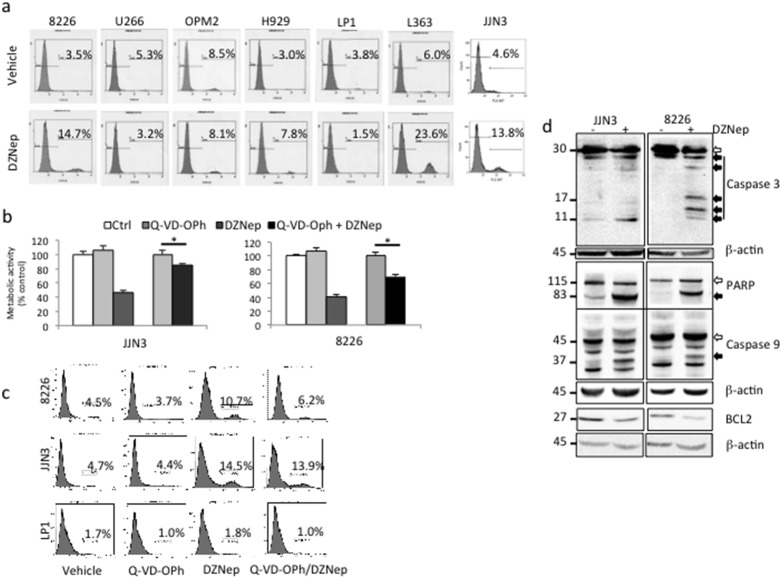
DZNep-induced cell death is apoptosis. (**a**) Exponentially growing MM cells were either treated with vehicle (DMSO 0.1%) or DZNep 1 µM for 72 h. Apoptosis was analyzed by FACS after APO2.7 staining. At least, 2×10^4^ events were gated. The percentage of apoptotic cells (stained for APO2.7) is indicated on the graph. (**b**) Exponentially growing JJN3 and 8226 cells were either pretreated with vehicle or Q-VD-OPh 10 µM for 1 h then with vehicle or DZNep 1 µM for 48 h. Cell proliferation was estimated by a MTS assay. Control samples referred to 100%. Here is shown a representative example from three independent experiments; each culture condition being in triplicate. Histograms show mean ± SD, *p<0.05. (**c**) 8226, JJN3 and LP1 cells were treated with vehicle or Q-VD-OPh (10 µM for 1 h) and/or treated with DZNep (1 µM for 72 h). Cells were then stained with anti-APO2.7 Ab and analyzed by FACS. At least, 2×10^4^ events were gated. The percentage of apoptotic cells (stained for APO2.7) is indicated on the graph. (**d**) Western blots were performed with the indicated antibodies to study the caspase cascade; β-actin Ab was used as control of charge and transfer. White arrows show proforms of PARP and caspase 3/9 and black arrows the cleaved (cl.) and activated forms of proteins.

However, we observed that DZNep induced morphological changes in the responding 8226 cells. On images of transmission electronic microscopy, DZNep-treated 8226 cells adopted an “epithelioid” morphology (Figure 2a). Moreover, compared to LP1, 8226 cells tended to increase in size, at the perimeter, area and circularity levels (Figure 2b). These morphological modifications being compatible with prenecrosis swelling, we hypothesized that another type of cell death could be implicated, possibly necroptosis. ROS production is an essential event of necrosis-oriented cell death [27]. MMCLs were then treated with DZNep and the production of ROS was measured by FACS using the CM-H_2_DCFDA dye. DZNep did not induce ROS production in L363 cells (Figure 2c) or LP1 cells (not shown). *TXNIP* is a key regulator of ROS generation and has been previously described to be upregulated by DZNep treatment in acute myeloid leukemia [28]. We found that DZNep did not lead to significant changes in *TXNIP* expression in responsive or resistant MM cells (Figure 3d). Taken together, our results show that the cell death triggered by DZNep in MM is neither autophagy nor necroptosis.

### DZNep induces caspase-dependent and -independent apoptotic cell death

MMCLs were treated (vehicle or 1 µM DZNep for 72 h) and stained with PI or anti-APO2.7-phycoerythrin (PE) Ab before flow cytometry sorting. As shown Figure 3a, analysis of APO2.7-positive cells demonstrated that sensitive MMCLs (8226, L363 and JJN3) underwent apoptosis after drug treatment. The triggering of apoptosis by DZNep was confirmed by the appearance of a sub-G1 phase (*i.e*. containing apoptotic cells) for PI-stained cells (data not shown). DZNep-resistant cells (U266, OPM2 and H929) were not blocked at any phase of the cell cycle excluding an entry in quiescence/senescence. In agreement, in DZNep-treated cells, we did not observe the upregulation of quiescence/senescence-associated proteins p16 and p21 (data not shown). The use of a pan-caspase inhibitor (Q-VD-OPh) restored, albeit not completely, the proliferation of JJN3 and 8226 cells after treatment (1 µM DZNep or vehicle for 24 h) as assayed by the MTS assay (Figure 3b). After APO2.7 staining of DZNep-treated sensitive 8226 and JJN3 cells and resistant LP1 cells as control, we observed that Q-VD-OPh did not abrogate completely DZNep effects (Figure 3c). We concluded that the apoptosis triggered by DZNep proceeded by both caspase-dependent and -independent mechanisms. We, finally, performed Western blotting to observe the effects of DZNep on the caspase cascade. As exemplified Figure 3d, after DZNep treatment (72 h, 1 µM), caspase-3 and -9 were activated leading to PARP cleavage. Moreover, associated with the recruitment of the intrinsic apoptotic pathway, we observed the decrease of the anti-apoptotic protein BCL2 necessary for MM cells to enter apoptosis.

### EZH2 expression level is reduced by DZNep treatment in MMCLs but not its targets

**Figure 4:**
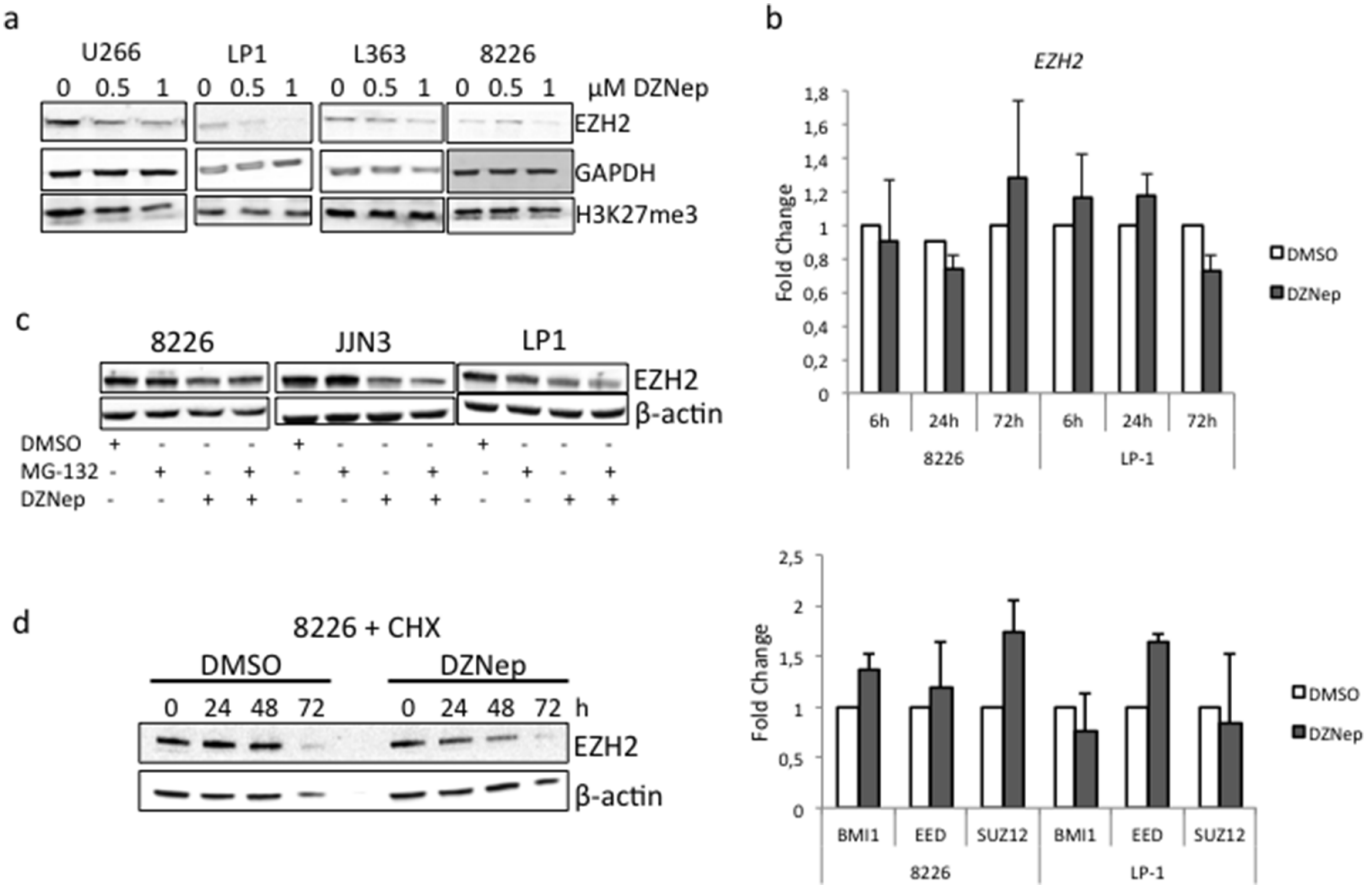
EZH2 expression is reduced at post-transcriptional level but is not involved in MM cells response to DZNep treatment. (**a**) MMCLs were treated with either vehicle (0) or indicated concentrations of DZNep for 72 h. Western blots were performed with indicated antibodies. Anti-GAPDH Ab was used for loading and transfer control. The experiment has been repeated three times. (**b**) Responsive 8226 and resistant LP1 cell lines were treated for different time intervals (**upper part**) or 72 h (**lower part**). The transcriptional expression of *EZH2* (**upper part**) or *BMI1*, *EED* and *SUZ12* (**lower part**) was studied by qRT-PCR using the ΔΔC_t_ method with *RPLP0* as internal standard. The fold change was calculated as 2^−ΔΔ*Ct*^. Indicated values corresponded to the mean ± SD from at least three independent experiments. (**c**) The responsive 8226 and JJN3 and the resistant LP1 cells were treated with vehicle, MG-132 (100 nM for 24 h), DZNep (1 µM for 24 h) or both. Cell were harvested; total proteins were purified, separated by SDS-PAGE and analyzed by Western blot with the indicated Abs. (**d**) 8226 cells were treated with CHX (100 µM for 1 h) then with vehicle or DZNep (1 µM for 24 h). Total proteins were purified, separated by SDS-PAGE and analyzed by Western blot with the indicated Abs.

Some previous reports have shown that DZNep-induced cell death results from the reduction of EZH2 expression level due to its degradation by the proteasome, and in turn, from the reduction of the H3K27me3 expression level [13], [14], [29], [30]. The levels of EZH2 and H3K27me3 were analyzed by Western blots in treated cells (DZNep 1 µM or vehicle for 72 h). The level of EZH2 protein decreased in a dose-dependent manner but to the same extent in resistant (U266, LP1, H929) and sensitive (L363, JJN3, 8226) cells (Figure 4a and not shown). Moreover, we did not detect any alteration of H3K27me3 levels in those cell lines (Figure 4a). This suggests that the triggering of apoptosis by DZNep is independent of EZH2 level and the global methylation status of H3K27 in MM cells. To further investigate the mechanisms of DZNep-induced EZH2 depletion, we analyzed the expression of *EZH2* gene as well as genes coding for PRC2 (*SUZ12*, *EED*) or PRC1 (*BMI1*) components by real-time reverse-transcription polymerase chain reaction (RT-PCR) after DZNep treatment (1 µM, 48 h). As shown Figure 4b, DZNep treatment did not lead to any significant variation of the transcription of those genes in good agreement with a previous report [14]. This implies that DZNep acted on EZH2 expression at a post-transcriptional level. MMCLs were next pretreated with the proteasome inhibitor MG-132 (100 nM for 24 h or vehicle) then treated with either DZNep 1 µM or vehicle for 48 h and the level of EZH2 analyzed by Western blot. As shown Figure 4c, the inhibition of the ubiquitin/proteasome degradation pathway did not stabilize EZH2 in DZNep-treated cells. We next analyzed the turnover of EZH2 protein in cells in which protein synthesis was inhibited by CHX. As shown Figure 4d, the level of EZH2 decreased faster in DZNep-treated cells than in vehicle-treated cells. We concluded that DZNep affects EZH2 synthesis rather than degradation. In conclusion, following DZNep-treatment, EZH2 level is regulated at a post-transcriptional level but not by the proteasome degradation pathway. We suggest from those data that the anti-survival properties of DZNep in MMCLs could be due to an off-target effect.

### DZNep impairs tumor cells engraftment and growth in their niche in NSG mice

**Figure 5:**
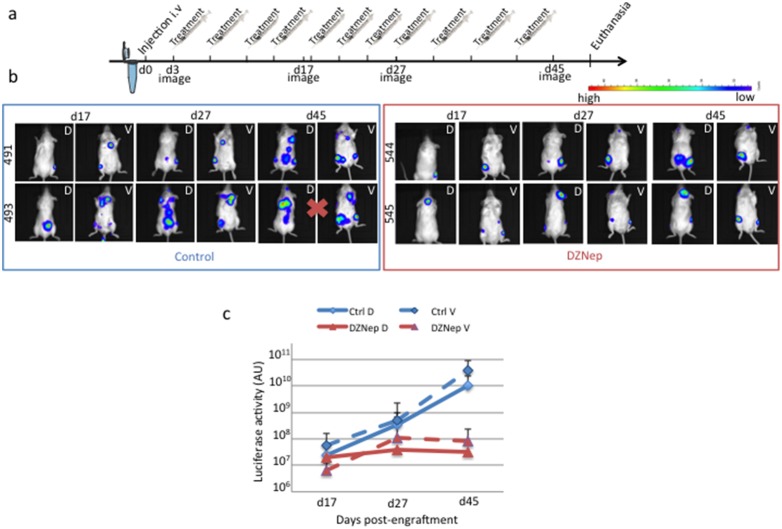
DZNep delays the engrafment and impairs the growth of MM cells in NSG mice. (**a**) RPMI 8226-GFP-Luc cells were injected into the caudal vein of NSG mice (n = 10) at day 1 (5×10^6^ cells were injected per animal). Three days later, mice were separated into two groups (n = 5 in each group), one received vehicle for control; the other was treated with 100 µg DZNEp twice a week as indicated in the scheme. At day 48, mice (except two, see below) were euthanized and tumors in soft tissues and bones removed for HES and IHC analyses. (**b**) BLI of the dorsal (D) and the ventral (V) sides of mice were taken at four sequential time points from day 3 to day 45. Both ventral and dorsal images of two mice in each group (mice #491/493 and #544/545) are shown. Mice 492 and 493 (red cross) showing hind leg paralysis were killed at day 36. (**c**) The luciferase activity of RPMI 8226-GFP-Luc cells in vehicle- (blue curves) and DZNep-treated (red curves) mice were determined into the two groups by BLI at the dorsal (plain line) and ventral (dotted line) levels.

**Figure 6:**
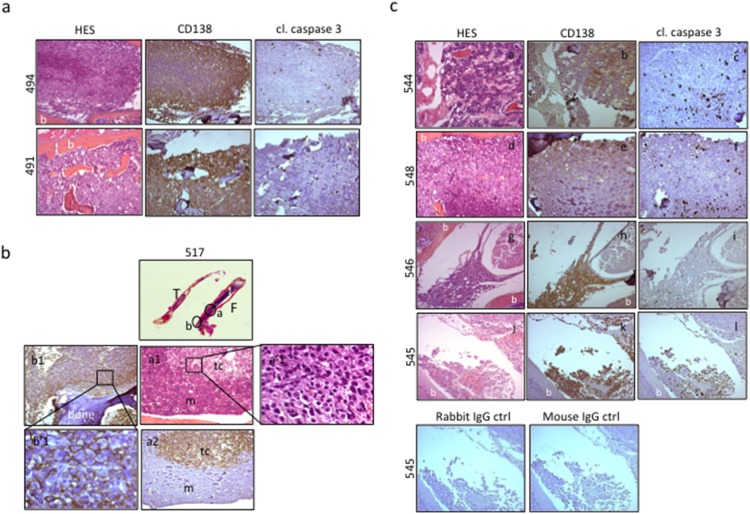
DZNep impairs the growth of MM cells in their niche. (**a**) Bones from control mice were processed and analyzed by HES and by IHC for CD138 and cleaved (cl.) caspase 3 staining. Images (x100 magnification) obtained for the femur of mouse #494 and the rachis of mouse #491, both vehicle-treated. CD138-positive cells that invaded bone marrow, caused bone (b) disorganization within the femur (#494) and the intervertebral discs (#491). Concomitantly, few cl. caspase 3 staining was noticed. (**b**) The femur (F) and tibia (T) from vehicle-treated mouse #517 were processed, scanned (x30 magnification), HE stained and analyzed for CD138 labeling by IHC (x100 magnification, a1, a2, b1; x630 magnification, a’1, b’1). Foci (a, b, c, circled regions) of typical CD138-membrane stained (b’1) MM cells tumors cells (tc) are visible within disorganized mouse bone marrow (m). MM cells mainly concentrated in trabecular areas (b, b1, b’1) but also in delineated medullae foci (a, a1, a’1, a2). (**c**) Examples of histological analyses (HES, CD138 and cl. caspase 3 staining) from rachis (mice #544 and 546), femur (mouse #548) and skull (mouse #545) samples removed from DZNep-treated series. CD138-positive *bona fide* MM cells invaded bone (b) tissues causing destruction and disorganization (puddles or ghosts associated with elevated caspase 3 activity). This high caspase 3 activity underlined DZNep therapeutic efficacy. Images of negative isotype rabbit or mouse IgG controls done on skull sections from mouse 454 are shown.

To assess the activity of DZNep *in vivo*, we used the RPMI 8226-GFP-Luc cell line capable to graft into immunodeficient mice and to progress as myeloma tumor. Tumor localization and growth could be monitored by non-invasive BLI. Moreover, this model has been found pertinent for the evaluation of therapies in multiple myeloma [23]. We first confirmed that RPMI 8226-GFP-Luc cell line responded as well as the parental cell line to DZNep (data not shown). We injected RPMI 8226-GFP-Luc cells in the caudal vein of NSG mice. Two mouse groups were designed: the control one was injected with vehicle (n = 5), the other received DZNep (n = 5) as scheduled in Figure 5a. Two days post-injection, areas of luciferase activity were detected mainly in the lungs and at the basis of the tail in all mice (data not shown). As soon as three days post-injection, mice were then treated (DZNep or vehicle) twice a week and sequentially imaged as depicted in Figure 5a. In agreement with published results [23], in vehicle-treated mice, RPMI 8226-GFP-Luc cell line progressed as a myeloma tumor predominantly in the bone marrow. Foci of luciferase activity were present in the limbs (both anterior and posterior), pelvic region, skull, sternum, ribs and spinal vertebrae (Figure 5b and Table 1). The pattern of luciferase foci varies from one mouse to the other but was specific for each one (Figure 5b). MM growth in the bones resulted in hind legs paralysis in two out five mice in the group of vehicle-treated animals (mice #494 and 495); they were sacrificed at day 36. HES and CD138 detection by IHC further confirmed the presence of myeloma cells in the bone marrow (Figure 6a). Low levels of activated caspase 3 were present in control mice (Figure 6a). Considering the DZNep-treated group, the growth of MM tumors was delayed in two out of five mice (mice #548 and 549). At day 27, tumor localizations were the same than in the vehicle-treated group confirming that soft tissues were not the primary sites for MM engraftment (Table 1). Indeed, only two out of five mice in each series showed luciferase activity in the abdomen and/or pelvic regions. We further characterized these cells to be CD138^+^ myeloma cells having invaded lymphatic nodes (not shown). Again the pattern of luciferase foci was specific for each mouse (Figure 5b). Importantly, in the DZNep-treated group, the ventral and dorsal luciferase activities were lower than in the vehicle-treated group. Moreover, at day 27, tumors stopped growing (Figure 5c). HES and CD138 staining confirmed the presence of myeloma cells in the bones of limbs, rachis and skull of animals (Figure 6b and c). As exemplified Figure 6b, tumor cells concentrated in well-defined foci within the femur (F) and the tibia (T) of mouse 517. Bones were invaded by CD138^+^ tumoral cells (tc) with large and dense nuclei among a mixture of mouse hematopoietic cells (m). Although the intensity of cl. caspase 3 staining varied from one sample to the other (compare the insets (c), (f) and (i), Figure 6c), caspase 3 activity was associated with CD138 staining within disorganized bone marrow. Due to the destruction of bone and bone marrow structures caused by IHC protocols, we were unable to quantify the level of activated caspase 3. However, the analysis of several sections from various bones and various animals confirmed higher caspase 3 activities in DZNep-treated series (compare Figure 6a and c). Our data demonstrated that, *in vivo*, DZNep delayed the engraftment and impaired the growth of MM cells in their physiological niche.

**Table 1:** Localization of tumoral foci in NSG mice.

Treatment	DMSO	DZNep	Global
Sternum	1/5	1/5	2/10
Rachis	3/5	2/5	5/10
Posterior limbs	5/5	4/5	9/10
Skull	0/5	2/5	2/10
Pelvic region	1/5	1/5	2/10
Abdomen	1/5	1/5	2/10

The distribution of tumoral cells was assessed by BLI in ten mice (five per group), 45 days after the inoculation of 5×10^6^ RPMI 8226-GFP-Luc cells in the caudal vein of immunodeficient NSG mice.

### DZNep shows specificity towards CD138^high^ MM cells

**Figure 7:**
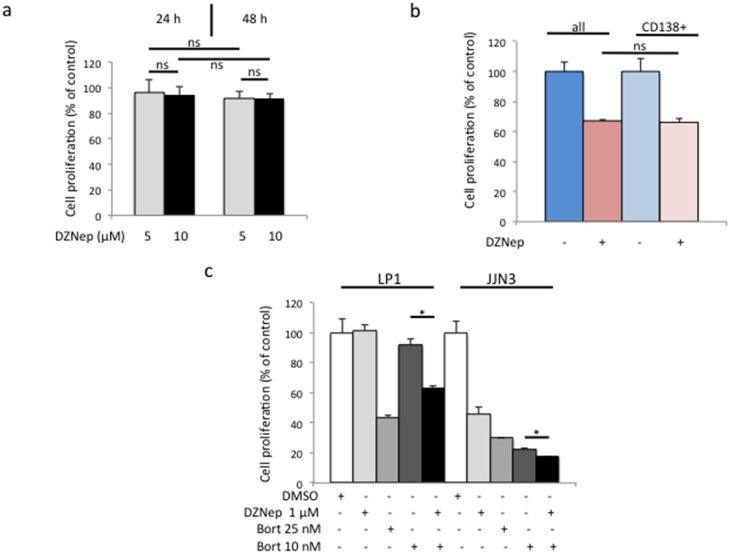
DZNep kills CD138^+^ MM cells and co-operates *in vitro* and *in vivo* with bortezomib. (**a**) Stromal HS-5 cells were seeded in 96-well plates at the density of 10^4^ cells/well and cultured for three days. Vehicle or DZNep (1 µM) was further added in five wells per culture condition and plates incubated for 24 and 48 h. Cell viability was determined by a MTS assay. On the graph are the means and SD values. ns, not significant. (**b**) Exponentially growing 8226 cells were separated into CD138^low^ and CD138^high^ populations. The sensitivity of CD138^high^ population and global population to DZNep (1 µM for 72 h) was compared by the MTS assay as before. ns, not significant with the Student’s *t*-test. (**c**) LP1 DZNep-resistant cells and JJN3 -sensitive cells were either treated with vehicle or DZNep 1 µM or bortezomib (Bort) 10–25 nM for 24 h or sequentially first with Bort then DZnep for 48 h. Cell proliferation was estimated by the MTS assay. Control samples referred to 100%. Here is shown a representative example from three independent experiments; each culture condition being in triplicate. Histograms show means ± SD, *p<0.05.

IHC analysis of bone sections in DZNep-treated mice showed a dramatic disorganization of bone structures (Figure 6c). This could be due to the death of stromal cells after DZNep-treatment or by the disappearance of apoptotic tumor cells that invaded bone matrix. In aiming to verify this point, we assessed the cytotoxicity of DZNep on HS-5 human stromal cells. As shown Figure 7a, DZNep, tested as before on MMCLs, had no effect on cell viability and proliferation. We concluded that DZNep acts specifically on MM cells and not on their bone marrow microenvironment.

We, and others, have characterized a sub-population of CD138^low^ cells within several MM cell lines, including 8226 cells, having the properties of cancer stem cells [20], [31]. Recently, it has been reported that those clonogenic cells show an enriched pattern of stemness genes including PRC genes and could be more sensitive than CD138^high^ cells to DZNep [32]. We analyzed the response of CD138^high^ cells purified from 8226 cells towards DZNep treatment (1 µM). As shown Figure 7b, the sensitivity of CD138^high^ cells towards DZNep was the same than the global population 72 h post-treatment leading us to conclude that CD138^high^ MM cells are able to undergo apoptosis when treated with DZNep.

### DZNep co-operates with bortezomib to kill MM cells *in vivo* and *in vitro*

**Figure 8:**
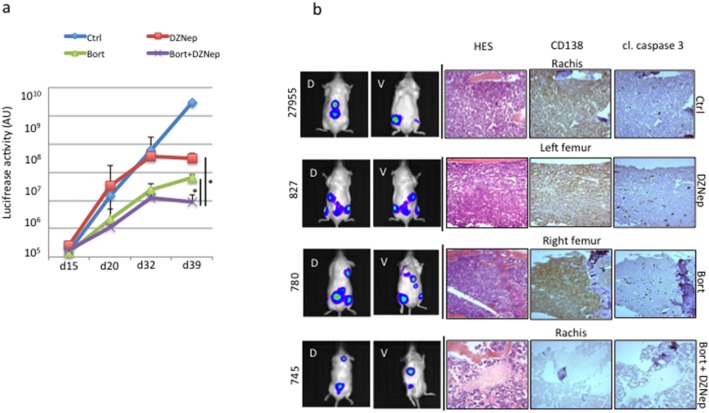
DZNep co-operates *in vivo* with bortezomib. (**a**) RPMI 8226-GFP-Luc cells were injected into the caudal vein of NSG mice (n = 20) at day 1 (5×10^6^ cells were injected per animal). Ten days later, mice were separated into four groups (n = 5 in each group), one received vehicle for control, one was treated with 12.5 µg bortezomib i.p. twice a week, one was treated with 50 µg DZNep i.p. every two days, one was treated with bortezomib plus 50 µg DZNep i.p. every two days. Mice were imaged at days 15, 20, 32 and 39. At day 40, all mice (except three, see below) were euthanized. Two mice in the bortezomib-treated group and one in the bortezomib plus DZNep group died at day 32. BLI of the dorsal and the ventral sides of mice was taken at these time points and added. The luciferase activity (in arbitrary unit) representative of tumor growth in each series (mean ± SD) is represented in the graph; in blue the control group, in red the DZNep-treated group, in green the bortezomib-treated group and in purple the bortezomib/DZNep group. *p<0.05 with the Student’s *t*-test. (**b**) Examples of histological analyses (HES, CD138 and cl. caspase 3 staining) from rachis (mouse #27955 from control group and mouse #745 from DZNep/borezomib group), left femur (mouse #827) from DZNep-treated group, and right femur (mouse #780) from bortezomib group. CD138-positive MM cells invaded bone tissues causing destruction and disorganization. In DZNep- or bortezomib-treated animals a high caspase 3 activity underlined therapeutic efficacy. Impressively, in mice #745 treated by both compounds, little CD138-positive cells were detected suggesting a possible cure.

Combined therapies show superior efficacy in myeloma patients [33]. We analyzed the response of JJN3 and LP1 cells exposed *in vitro* to bortezomib, largely used in clinical practice, alone or in combination with DZNep. As expected, JJN3 and LP1 cells responded to bortezomib treatment (10 and 25 nM for 24 h) and were sensitive or resistant to DZNep treatment (1 µM for 24 h), respectively (Figure 7c). Importantly, we observed additive effects when cells were cotreated with drugs even in the resistant LP1 cells, indicating that bortezomib overcomes DZNep resistance. The additivity of combined treatment on apoptosis was further confirmed on DZNep- and/or bortezomib-treated cells stained with the anti-APO2.7 Ab and analyzed by cytometry (Table 2). We finally assessed the combined bortezomib/DZNep treatment *in vivo* in the same settings than before. As previously reported [34], [35], bortezomib was very efficient and inhibited tumor growth as soon as 20 days post-treatment (Figure 8a). In agreement with *in vitro* results, combined bortezomib plus DZNep treatment exerted higher growth inhibition towards 8226 xenografts but only at the end of the experiment (day 39, Figure 8a). We stopped the experiment at that time because of bortezomib toxicity in mice. IHC confirmed the co-operation between DZNep and bortezomib to kill MM cells (Figure 8b).

**Table 2:** Additivity of DZNep and bortezomib proapoptotic effects on MM cells.

Cell line	Treatment	APO2.7+cells (%)
JJN3	Vehicle	4.3
	DZNep 1 µM	11.2
	Bortezomib 10 nM	43.7
	Bortezomib 25 nM	50.4
	DZNep 1+Bortezomib 10	54.9
	DZNep 1+Bortezomib 25	55.6
LP1	Vehicle	4.5
	DZNep 1 µM	1.9
	Bortezomib 10 nM	43.0
	Bortezomib 25 nM	54.9
	DZNep 1+Bortezomib 10	50.9
	DZNep 1+Bortezomib 25	55.4

JJN3 DZNep-sensitive cells and LP1 DZNep-resistant cells were either treated with vehicle or DZNep 1 µM or bortezomib (Bort) 10–25 nM for 48 h or sequentially first with bortezomib for 24 h then DZnep for 24 h. Apoptosis was estimated after anti-APO2.7 staining of cells and cytometry sorting. At least, 2×10^4^ events were gated.

## Discussion

The DZNep was first studied to enlarge the antiviral drugs arsenal and was further shown to induce cancer cell death [13]. DZNep became a promising anti-tumoral drug with a significant efficacy on various cell types and no evident toxicity *in vivo* [14], [36], [37]. Although DZNep is an inhibitor of AdoHcy, in most of cancers it acts through the reduction of EZH2 level and in turn, the re-expression of genes silenced by PRC2 *via* the demethylation of K27H3 [13], [14], [37]. Since MM patients exhibit a gene signature enriched for H3K27me3 marks and in particular in PRC2-silenced genes [10], the targeting of PRC2 components could be relevant for treatment. We show here that DZNep induces caspase-dependent and -independent apoptosis in a subset of MM cells *in vitro* and *in vivo*, alone or in combined therapy, but the induction of cell death is not mediated by the downregulation of EZH2 and the subsequent re-expression of PRC2-silenced genes. One mechanism of EZH2 depletion is its degradation by the ubiquitin/proteasome degradation pathway [13], [14], [29], [30]. However, we reported here that DZNep affects EZH2 synthesis rather than degradation suggesting that it may act through a global effect on cell metabolism as previously suggested [12].

The effects of DZNep on MM cell lines, primary cells and *in vivo* models have been reported previously in two contrasting reports. In the first one, the reactivation of silenced genes by the pharmacological depletion of EZH2 by DZNep allows the induction of apoptosis in two MMCLs and in the 5T33MM *in vivo* model [10]. By contrast, in another study, in agreement with our data, the induction of apoptosis in responsive MM cells was accompanied by the downregulation of EZH2 but not H3K27me3 [12]. Since, DZNep is a global inhibitor of histone methylation and displays no selectivity, others epigenetic marks such as trimethylation of lysine 20 on histone H4 (H4K20me3), dimethylation of lysine 9 on histone H3 (H3K9me2), trimethylation of lysine 79 on histone H3 (H3K79me3) etc., could be modified as reported in breast cancer cells [13]. DZNep treatment could induce modifications of the methylation of histones without affecting the trimethylation of H3K27 and, in turn, modifications of the global transcription, maybe by involving other epigenetic marks we did not explore.

Our results suggest that the decrease of EZH2 is probably not decisive for the apoptotic response of MM cells towards DZNep. We used siRNA to specifically induce the downregulation of *EZH2* and in turn, study the effect of decreased EZH2 on cell death. For an unknown reason, we did not obtained more than 50% gene extinction with reasonable cell viability. In those experimental conditions, cell survival was not affected (data not shown). Those data underlie a probable off-target effect for DZNep. This cellular mechanism of action should be investigated further but we know from previous results that DZNep could modify deeply cell metabolism and in particular, lipid biosynthesis [12].

Xie *et al*., reported previously no association between the sensitivity towards DZNep and the presence of the chromosomal translocation t(4;14) in MM cells [12]. In the subgroup of MM cells with t(4;14), the *MMSET* histone methyltransferase is overexpressed and in turn, the global pattern of histone methylations is modified [38]. Interestingly, in our study, all responsive cells (8226, JJN3, L363 and EJM) carry a translocation that affects a gene coding for a transcription factor of the MAF family (*c-MAF* for t(14;16) or *MAFB* for t(14;20)). This is of particular interest, since these translocations are associated with adverse prognosis in clinical practice [38]. We hypothesized that *MAF* could be directly impacted by DZNep treatment. However, it has become unlikely since the level of cyclin D2, a direct target of MAF, was not modulated after DZNep treatment as observed by RT-PCR and Western blot experiments (data not shown). Moreover, the overexpression of *MAF* transcripts is a frequent event even in MM cells which do not carry the t(14;16) translocation [39]. And c-MAF/MAFB proteins are present in the non-responsive LP1 and OPM2 cells [40]. Nevertheless, if a direct role of MAF can be excluded, the genetic background that accompanies t(14;16) or t(14;20) translocations could be important for sensitivity towards DZNep. The *TP53* gene status regulates the sensitivity of gastric cancer cells to DZNep [41]. DZNep depleted EZH2 in almost all tested cell lines but only those with a wild-type *TP53* are sensitive and growth-inhibited. In our series, *TP53* is abnormal in three responsive cells (8226, JJN3 and L363) ruling out a common mechanism of action for gastric cancer and myeloma cells.

In prostate cancer cells, DZNep has been shown to induce specifically the death of cancer stem-like cells (CSCs) [36]. EZH2 seems essential for glioblastoma cancer stem cell maintenance [42]. This is consistent with the key role of PRC in the commitment and differentiation of normal stem cells. A recent report described a greater efficacy of DZNep on MM CSCs having a CD138^low/−^ compared to CD138^high/+^ MM cells [32]. This constituted a supplemental argument for the epigenetic mode of action of the molecule. We have not directly assessed the response of CD138^low/−^ MM cells towards DZNep, but we showed here that MM cells expressing CD138 display similar sensitivity than the whole population. The precise phenotype of MM CSC or tumor-initiating cell is still debated. Recently, it was reported that exists a pool of CD19^−^CD138^+^ and CD19^−^CD138^−^ cells with an interconvertible phenotype that are functionally equivalent, having clonogenic properties and capable to propagate MM tumor *in vivo* [43]. From our data we can speculate that both cell types are sensitive towards DZNep. This implies that contrary to most anti-myeloma drugs, DZNep will be able to eliminate all the cell types constituting the tumors.

DZNep is a promising tool for cancer therapy. It induces the apoptosis of cancer cells but not of their normal counterparts: epithelial cells and fibroblasts [14] or more importantly hematopoietic cells [44]. We show here that DZNep does not trigger the death of stromal cells surrounding tumor cells in the bone marrow. Importantly, DZNep and bortezomib co-operate to eliminate *in vitro* and *in vivo* MM cells. The response of MM cells to DZNep deserves further exploration however our data suggest that lower doses of bortezomib, which have adverse effects in patients, could be administered in association with DZNep in patients with bad prognosis or relapse.
